# Y and ZSM-5 Hierarchical Zeolites Prepared Using a Surfactant-Mediated Strategy: Effect of the Treatment Conditions

**DOI:** 10.3390/ma17174401

**Published:** 2024-09-06

**Authors:** Andrea Ruggiu, Ana Paula Carvalho, Elisabetta Rombi, Angela Martins, João Rocha, Pier Parpot, Isabel C. Neves, Maria Giorgia Cutrufello

**Affiliations:** 1 Department of Chemical and Geological Sciences, University of Cagliari, Complesso Universitario di Monserrato, S.S. 554, 09042 Monserrato, CA, Italy; andrea.ruggiu@unica.it (A.R.); rombi@unica.it (E.R.); 2 National Interuniversity Consortium of Materials Science and Technology (INSTM), Via Giusti 9, 50121 Florence, Italy; 3 Centro de Química Estrutural, Institute of Molecular Sciences, Universidade de Lisboa, Campo Grande, 1749-016 Lisboa, Portugal; apcarvalho@fc.ul.pt (A.P.C.); amartins@deq.isel.ipl.pt (A.M.); 4 Departamento de Química e Bioquímica, Universidade de Lisboa, Campo Grande, 1749-016 Lisboa, Portugal; 5 Departamento de Engenharia Química, Instituto Superior de Engenharia de Lisboa, Instituto Politécnico de Lisboa, Rua Conselheiro Emídio Navarro, 1959-007 Lisboa, Portugal; 6 Departamento de Química and CICECO, Universidade de Aveiro, 3810-193 Aveiro, Portugal; rocha@ua.pt; 7 CQUM—Centre of Chemistry, Chemistry Department, University of Minho, Campus de Gualtar, 4710-057 Braga, Portugal; parpot@quimica.uminho.pt (P.P.); ineves@quimica.uminho.pt (I.C.N.); 8 CEB—Centre of Biological Engineering, University of Minho, Campus de Gualtar, 4710-057 Braga, Portugal

**Keywords:** Y zeolite, ZSM-5 zeolite, hierarchical zeolites, surfactant-mediated alkaline treatment, mesoporosity, hydrothermal treatment duration, ZSM-5 pretreatment

## Abstract

Diffusional limitations associated with zeolite microporous systems can be overcome by developing hierarchical zeolites, i.e., materials with a micro- and mesoporous framework. In this work, Y and ZSM-5 zeolites were modified using a surfactant-mediated hydrothermal alkaline method, with NaOH and cetyltrimethylammonium bromide (CTAB). For Y zeolite, after a mild acidic pretreatment, the effect of the NaOH+CTAB treatment time was investigated. For ZSM-5 zeolite, different concentrations of the base and acid solutions were tested in the two-step pretreatment preceding the hydrothermal treatment. The properties of the materials were studied with different physical–chemical techniques. Hierarchical Y zeolites were characterized by 3.3–5 nm pores formed during the alkaline treatment through the structure reconstruction around the surfactant aggregates. The effectiveness of the NaOH+CTAB treatment was highly dependent on the duration. For intermediate treatment times (6–12 h), both smaller and larger mesopores were also obtained. Hierarchical ZSM-5 zeolites showed a disordered mesoporosity, mainly resulting from the pretreatment rather than from the subsequent hydrothermal treatment. High mesoporosity was obtained when the concentration of the pretreating base solution was sufficiently high and that of the acid one was not excessive. Hierarchical materials can be obtained for both zeolite structures, but the pretreatment and treatment conditions must be tailored to the starting zeolite and the desired type of mesoporosity.

## 1. Introduction

Zeolites are highly crystalline aluminosilicates with a peculiar microporous system, characterized by high thermal and chemical stability, large surface area, and peculiar acid properties [1]. Thanks to their characteristics, zeolites are used in several fields as molecular sieves, chemical sorbents, heterogeneous acid catalysts, and supports for metal and metal-complex catalysts. Several types of zeolites, characterized by different structures and chemical properties, are known. Two of the most common and widely used are the Faujasite (FAU) Y and the ZSM-5 zeolite. Both these materials are commonly used as catalysts, particularly in petroleum refining [1] and in organic fine chemicals synthesis [2]. Their catalytic activity can also be exploited in the purification of wastewater [3]. Other applications can be the adsorption of contaminants from gas streams [4] or the preparation of membranes for the purification of organic compounds by pervaporation [5].

One of the most interesting features of these materials for heterogeneous catalysis is the shape and size selectivity granted by the narrow micropores, which are typically smaller than 1 nm [2,3,6]. However, the presence of such narrow micropores is also the main drawback of zeolites, since the diffusivity within the micropores is strongly limited for molecules with critical dimensions close to the micropore width. For this reason, several promising zeolite-based catalysts are not taken into consideration for further studies. The concern on this issue has increased in the past decade, with studies focused on enhancing the accessibility of the internal surface and active sites. One of the main approaches is the production of hierarchical zeolites, characterized by the presence of larger pores, generally mesopores, within the microporous system. Thanks to this peculiar framework, the diffusivity in the hierarchical zeolites is higher than in the microporous ones. The improvement in mass transfer can be exploited in catalytic, environmental, and biological applications, thanks to the enhancement in catalytic and adsorption performances [7]. When large molecules are involved, the use of hierarchical zeolites allows processes where reactants and/or products hardly diffuse in the typical microporous zeolite framework. The improved mass transfer is particularly useful in industrial catalytic processes (e.g., industrial hydrocracking, isomerization of organic compounds, fine chemicals synthesis) [8,9].

Hierarchical zeolites were originally obtained as a result of other postsynthetic modifications, such as desilication or dealumination [8,9,10]. Since then, two types of strategies have been proposed for the production of these materials: bottom-up, which consists of modified synthetic protocols for inducing the formation of mesopores, generally with hard or soft templating agents; top-down, in which the zeolite structure is modified postsynthesis, with desilication, dealumination, or surfactant-based procedures [7,8].

The surfactant-based method was developed for the first time by García-Martínez et al. [8,11,12] and consists of a hydrothermal alkaline treatment in the presence of a surfactant under autogenous pressure, preceded by a pretreatment necessary for fragilizing the structure. The main feature of this procedure is the development of an ordered mesoporosity, at variance with the random one obtained with the standard desilication or dealumination procedures. Thereafter a certain number of studies focused on the optimization of the conditions to obtain hierarchical zeolites with a tuned mesoporosity. Mendoza-Castro et al. [13] reviewed the surfactant-mediated alkaline method, starting from the original procedure for FAU zeolites with NH_4_OH and cetyltrimethylammonium bromide (CTAB), preceded by a citric acid pretreatment. They described the effect of the treatment conditions (temperature and duration, base and surfactant concentration) and gave further insight into the application to other zeolite structures, such as Beta (BEA) and Mordenite (MOR). Modification of ZSM-5 zeolites with NH_4_OH and CTAB—preceded by a treatment involving first a base and subsequently an acid—was also proposed [14]. Some studies also explored the effect of the use of alternative bases (NaOH or tetrapropylammonium hydroxide) [15,16] and of other surfactants with different chain lengths [16,17] for obtaining hierarchical Y zeolites.

The interest in the modification of conventional zeolites by a surfactant-mediated alkaline procedure is increasing, thanks to the possibility of enhancing the catalytic performance in valuable processes such as hydrocarbon cracking, cyclohexane oxidation, and Friedel–Crafts acylation [14,15,17,18,19]. In one of these studies [17], an HY zeolite was modified by the surfactant-mediated method, using NH_4_OH and either CTAB or dodecyl trimethylammonium bromide (DTAB), and the effect of the treatment time was studied. Recently, both hierarchical HY and ZSM-5 zeolites were obtained using NH_4_OH and CTAB [19], and the effect of the concentration of the surfactant and of the duration of the hydrothermal treatment was investigated. In the case of ZSM-5, the effectiveness of the pretreatment employed for preparing the zeolite structure for the surfactant-mediated hydrothermal step was found to be limited. Consequently, rather than coming from the formation of mesopores within the crystal structure during the surfactant-mediated hydrothermal treatment, the mesopores of the hierarchical ZSM-5 materials derived from the aggregation of the small crystals formed during the pretreatment (i.e., intercrystalline mesopores).

In this work, both Y and ZSM-5 zeolites were modified by the surfactant-mediated hydrothermal alkaline method. Starting from the procedure employed in a previous work [18], the same surfactant (CTAB) at a chosen concentration was used; as for the base, NaOH was chosen (instead of NH_4_OH, which was previously employed) at a concentration typically used by other authors [8]. For the Y zeolite in the present work, keeping constant all the other treatment and pretreatment parameters, the effect of the NaOH+CTAB treatment time was studied. Treatment durations ranging between short (2 h) and long (48 h) times were applied. Since in the case of the ZSM-5 zeolite the two-step procedure preceding the hydrothermal treatment had proved to be not effective enough [19], for this material the aim of the present work was finding more effective pretreatment conditions. Therefore, the use of higher concentrations of the solutions employed in both pretreatment steps was tested, whereas all the operating parameters of the hydrothermal treatment were kept constant.

## 2. Materials and Methods

### 2.1. Materials

Citric acid (C_6_H_8_O_7_, 99.5 wt%) was supplied by Sigma-Aldrich (Darmstadt, Germany). Cetyltrimethylammonium bromide (CTAB, C_19_H_42_BrN, >98 wt%), lithium tetraborate (Li_2_B_4_O_7_, 99.9 wt%), sodium hydroxide (NaOH, >98 wt%), and sulfuric acid (H_2_SO_4_, 98 wt%) were purchased from Merck (Darmstadt, Germany). Nitric acid (HNO_3_, 65 wt%) was supplied by Carlo Erba (Cornaredo, Italy). Nitrogen (N_2_, 99.99 wt%) was purchased from Air Liquide (Paris, France). NaY (CBV-100, SiO_2_/Al_2_O_3_ = 5.1 mol/mol) and NH_4_ZSM-5 (CBV 3024E, SiO_2_/Al_2_O_3_ = 30 mol/mol) zeolites were provided by Zeolyst International in powder form (Conshohocken, PA, USA).

### 2.2. Preparation of Hierarchical Zeolites

To obtain the hierarchical forms, the commercial NaY and NH_4_ZSM-5 zeolites were modified through a surfactant-mediated hydrothermal alkaline method derived from a procedure reported in previous works [17,19].

Prior to the hydrothermal treatment, the zeolites were submitted to different pretreatments. NaY was dispersed in a 10 wt% citric acid solution, using 2 mL of solution for 1 g of zeolite. After around 17 h under stirring at room temperature, the solid was recovered by filtration, washed with distilled water, and dried at 110 °C for one night. The pretreated sample was named Y_P. In the case of NH_4_ZSM-5, before the alkaline treatment, it was necessary to fragilize the structure in two consecutive steps: first the zeolite was dispersed in a stirred NaOH solution (0.25, 0.5, or 1 M) for 1 h at 80 °C and then recovered by filtration, washed with distilled water, and dried at 110 °C overnight. Subsequently, the resulting powder was dispersed in a stirred H_2_SO_4_ solution (0.6 or 1.2 M) for 3 h at 80 °C and finally recovered by filtration, washed with distilled water, and dried at 110 °C for one night; the solid submitted to such two-step pretreatment was named ZSM-5_P_*x*/*y*, where *x* and *y* represent the molar concentrations of the NaOH and H_2_SO_4_ solutions, respectively.

The Y_P and ZSM-5_P_*x*/*y* samples were then used to prepare the hierarchical structures through the surfactant-mediated hydrothermal alkaline treatment. Typically, 0.39 g of zeolite was added to 25 mL of a 0.37 M NaOH solution containing 0.27 g of CTAB (0.03 M). After stirring for 20 min at room temperature and adjusting the pH to 11 with a 2 M HCl solution, the dispersion was transferred into a 50 mL PTFE-lined stainless-steel autoclave and heated at 150 °C under autogenous pressure for 2, 6, 12, 24, or 48 h (only for 6 h in the case of the ZSM-5_P_*x*/*y* samples). The obtained solid was then filtered, washed with distilled water, and dried overnight at 110 °C. The removal of the surfactant was achieved by calcination of the solid at 550 °C for 4 h. The final calcined materials were labeled as Y_C*t* and ZSM-5_*x*/*y*_C*t*, where *t* indicates the duration (in hours) of the NaOH+CTAB hydrothermal treatment.

For most samples, different batches were prepared and the reproducibility of the procedure was always verified.

The main operating parameters used for obtaining the modified Y and ZSM-5 samples are summarized in Table 1 and Table 2, respectively.

### 2.3. Characterization

Inductively coupled plasma optical emission spectroscopy (ICP-OES) analyses were performed with a 5110 ICP-OES spectrometer (Agilent Technologies, Santa Clara, CA, USA) to quantitatively determine the Al and Si content. Each sample (*ca.* 0.05 g) was calcined at 500 °C for 12 h, mixed with lithium tetraborate (1:15 *w*/*w*), placed in a platinum crucible, and then fused at 1000 °C in a furnace for 30 min. After cooling of the melt, the resultant fusion bead was dissolved in 20 mL of a nitric acid solution (0.80 M) at 80 °C for about 30 min and finally diluted to the desired volume by type-1 water.

X-ray diffraction (XRD) analyses were performed on a PANalytical (Malvern, UK) PW3050/60X’Pert PRO diffractometer with θ/θ Bragg–Brentano geometry, equipped with an X’Celerator detector and using monochromatized Cu-Kα radiation as incident beam (40 kV-30 mA). The degree of crystallinity of the Y and ZSM-5 zeolites was calculated from the area of the peaks according to the ASTM 3906-03 and ASTM D5758-01 methods [20,21]. Peak integration was performed using Origin2021 software.

Textural analyses were carried out with an ASAP 2010 (Micromeritics, Norcross, GA, USA) by determining the nitrogen adsorption/desorption isotherms at −196 °C. Prior to analysis, the samples (*ca*. 0.07 g) were outgassed under vacuum (7.5 × 10^−5^ Torr) at 300 °C for 2 h. For the evaluation of the mesopore volume distribution the DFT method was used. The quantitative analysis of the micro- and mesoporosity was made by applying the α_s_-method [22], using the isotherms of a non-porous silica as reference.

Transmission electronic microscopy (TEM) was performed with a H-8100 transmission electron microscope (Hitachi, Chiyoda, Japan) operating at 200 kV. Before the analysis each sample was dispersed in ethanol and sonicated, then the dispersion was deposited onto TEM grids and dried in air.

The ^29^Si magic-angle spinning (MAS) nuclear magnetic resonance (NMR) spectra were recorded in a Bruker (Billerica, MA, USA) Avance III 400 NMR (9.4 T) wide-bore spectrometer at 79.5 MHz on a double-resonance 7 mm Bruker probe with a 5 kHz spinning rate, 45° radio-frequency (RF) pulses, and a 60 s recycle delay. The acquisition parameters for the ^29^Si cross-polarization (CP) MAS were: ^1^H and ^29^Si 90° pulses set to 2 and 6 ms, corresponding to an RF field strength of 125 and 42 kHz, respectively. The CP step was implemented using a contact time of 2 ms with a ramp shape in the ^1^H channel of 50–100% and ^29^Si RF field strength of 66 kHz. During the acquisition, SPINAL-64 decoupling with an RF field strength of 50 kHz (SPINAL basic pulse length of 9.5 ms) was employed. Chemical shifts are quoted in ppm from tetramethylsilane (TMS). The Q^n^ distribution was obtained by integrating the peaks with Origin2021 software for the Y samples and deconvoluting the NMR spectra with Fityk software (https://fityk.nieto.pl/, accessed on 9 July 2024) for the ZSM-5 samples. The Si/Al ratio was estimated using Equation (1):

(1) $$\frac{\mathrm{Si}}{\mathrm{Al}}=\frac{I}{\sum 0.25\cdot n\cdot I_{n}}\;,$$

where *I* is the total intensity of the ^29^Si MAS-NMR signals and $I_{n}$ is the intensity of the signal corresponding to the Q^n^ sites. The ^27^Al MAS-NMR spectra were recorded on a Bruker (Billerica, MA, USA) Avance III HD 700 MHz (16.4 T) narrow-bore spectrometer at 182.4 MHz on a double-resonance 4 mm Bruker probe with a 15 kHz spinning rate. Single quantum (“normal”) spectra were recorded using a short RF pulse of 0.27 μs (equivalent to a π/18 flip angle), calibrated on an aqueous solution of Al(NO_3_)_3_, with 100 kHz field strength and 0.5 s recycle delay.

## 3. Results and Discussion

### 3.1. Y Zeolites

In previous works [17,19] the hydrothermal surfactant-mediated alkaline method, using NH_4_OH and either CTAB or DTAB, was applied to the Y zeolite; after a mild acidic pretreatment, the effect of the duration of the hydrothermal treatment and of the surfactant concentration was investigated. The present Y materials were obtained, after the same pretreatment, using NaOH as the base and CTAB as the surfactant, keeping constant the solutions concentrations and changing the treatment time.

A Si/Al molar ratio of 2.4 was determined by ICP-OES analysis of the commercial NaY zeolite, in good agreement with the nominal value (Si/Al = 2.55, calculated from the SiO_2_/Al_2_O_3_ molar ratio value of 5.1 provided by the producer). After the hydrothermal treatment, whatever the duration, the ratio was found to be 3.5. This value was most likely due to the dealuminating effect of the acidic pretreatment.

**Figure 1:**
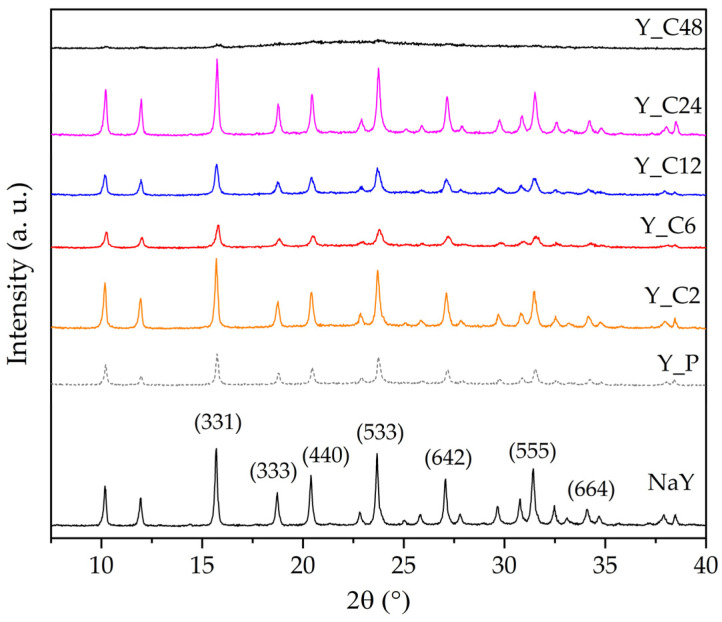
X-ray diffraction patterns of the Y zeolites. Miller indexes are attributed according to the IZA Database of Zeolite Structures (http://www.iza-structure.org/databases, accessed on 9 July 2024).

The XRD patterns of the starting and the modified Y zeolites are reported in Figure 1. The diffractograms of Y_P and of the samples treated up to 24 h show the typical structure of the FAU-type zeolites, pointing out that the crystalline structure is preserved, although significant differences in the intensity of the peaks can be observed. For the longest treatment time, the structure is practically completely destroyed, as indicated by the absence of significant reflections in the XRD pattern of the Y_C48 sample.

To quantify the loss in crystallinity due to the treatment, the degree of crystallinity (*C*_XRD_) was calculated from the area of the (331), (333), (440), (533), (642), (555), and (664) peaks [20], using the commercial NaY zeolite as the reference. The results are reported in Table 3 and show an interesting trend. The mild acidic pretreatment causes a loss in crystallinity (*C*_XRD_ = 72%). Then, when Y_P is submitted to the surfactant-mediated alkaline treatment, crystallinity either increases or decreases, depending on the duration of the treatment. This non-monotonic trend (Table 3) should derive from the complex interplay of the phenomena taking place during the treatment: NaOH attacks the crystalline structure, which, however, in the hydrothermal conditions, tends to reconstruct; CTAB forms aggregates, which can diffuse only in pores that are sufficiently wide; the destruction–reconstruction of the structure around such aggregates leads to the formation of mesopores. The relative rate of such processes would lead to different materials depending on how long the pretreated sample undergoes the surfactant-mediated alkaline treatment, the destruction of the zeolite structure definitively prevailing on the reconstruction only for a long-lasting procedure.

In effect, from the XRD results (patterns in Figure 1 and *C*_XRD_ values in Table 3) similar features can be observed for the samples obtained after intermediate treatment times (6 or 12 h), as well as—interestingly—for those obtained after short (2 h) or long (24 h) times.

**Figure 2:**
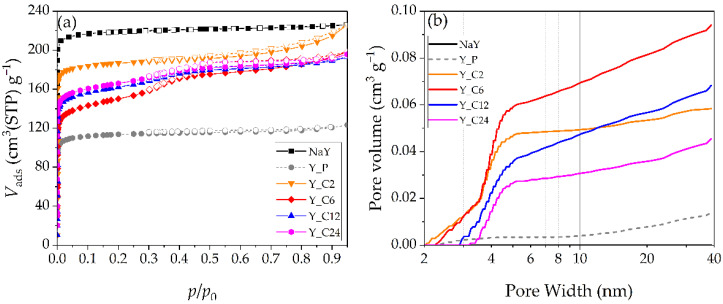
(**a**) N_2_ physisorption isotherms of the Y zeolites (adsorption: full symbols, desorption: open symbols); (**b**) Cumulative mesopore volume curves of the Y zeolites, obtained with the DFT method.

The N_2_ adsorption–desorption isotherms at −196 °C of the starting, the pretreated, and the hierarchical Y zeolites are reported in Figure 2a, whereas Figure 2b reports the cumulative pore volume curves obtained by applying the DFT method. The results of the quantitative analysis of the micro- and mesoporosity performed with the α_s_-method are summarized in Table 3, in terms of the volume of narrow micropores (ultramicropores, φ < 0.7 nm), larger micropores (supermicropores, 0.7 nm ≤ φ ≤ 2 nm), and mesopores (φ > 2 nm).

Both the NaY zeolite and the pretreated Y_P sample show type I isotherms, typical for microporous materials. Virtually no mesopores are present in the starting microporous NaY sample, as shown by its cumulative mesopore volume curve lying on the *x*-axis (Figure 2b). The simple acid treatment causes a loss in micropore volume without a significant formation of mesopores (Table 3 and Figure 2b). It is possible that part of the micropores have been blocked by some debris formed during this step.

The surfactant-mediated hydrothermal treatment of Y_P generally leads to the formation of supermicropores and mesopores and to an increase in the total pore volume.

After 2 h of treatment (Y_C2 sample), the isotherm is mostly of type I, but the upward deviation and the presence of a hysteresis loop at high pressure suggest the formation of interparticle meso- and/or macropores. The formation of small mesopores (2–5 nm) is actually revealed by the mesopore volume curve (Figure 2b), which also shows a very limited contribution of larger mesopores. Noteworthily, the micropore volume—in both ultra- and supermicropore ranges—also increases with respect to the pretreated sample Y_P (Table 3). The higher *V*_ultra_ value (0.21 cm^3^ g^−1^) could be due to the recovery of the original microporosity of the zeolite after the removal of the debris formed during the pretreatment and/or a partial reconstruction of the zeolite microporous structure. The formation of supermicropores, together with mesopores, is the effect of the hydrothermal alkaline treatment in its initial stage.

A 24 h treatment leads to a material with micro- and mesopore volume values similar to those of Y_C2 (Table 3), in agreement with the structure reconstruction revealed by XRD also for the Y_C24 sample. Nonetheless, significant differences between Y_C2 and Y_C24 materials can be observed in Figure 2. The adsorption isotherm (Figure 2a) is characterized by a small mid-pressure step and a hysteresis loop; it can then be classified as a type I+IV isotherm and indicates the presence of a microporous framework together with some mesopores. From Figure 2b it is apparent that the mesopore distribution has changed: besides a non-negligible contribution of mesopores larger than 5 nm, most of the mesopore volume is due to 3.3–5 nm pores, whereas smaller mesopores are no longer present. Thus, it emerges that the reconstruction observed after a 24 h treatment is accompanied by the formation of larger mesopores in comparison with those obtained after just 2 h.

When the surfactant-mediated alkaline treatment has an intermediate duration, the resulting materials (Y_C6 and Y_C12) show N_2_ adsorption isotherms characterized by the same features as that of Y_C24 (Figure 2a), the mid-pressure step being more visible for Y_C6. The mesopore volume distributions appear somewhat intermediate between those of Y_C2 and Y_C24 (Figure 2b). After 6 h of treatment the contribution of mesopores smaller than 3.5 nm is similar to that observed after 2 h, although the minimum size is a little larger (2.3 nm instead of 2 nm). When comparing the cumulative mesopore volume curves of Y_C12 and Y_C24, the dependence of the minimum mesopore width on the treatment time is further apparent.

Notably, although the materials obtained after intermediate times are also characterized by a significant contribution of larger mesopores, which corresponds to a high total mesopore volume calculated by applying the α_s_-method (Table 3), the common feature of all the hierarchical materials is the presence of 3.3–5 nm pores, which could be reasonably related to the structure reconstruction around the CTAB aggregates. At the concentration used in this work, the CTAB aggregates are expected to present an ellipsoidal worm-like shape with a minor axis of 2.4–2.6 nm [19]. The formation of supermicropores and mesopores, which after 2 h is just starting, becomes more important after 6 h, especially in the large mesopore range. This appears to happen at the expense of the original microporous structure, as suggested by the pore volume values reported in Table 3. Prolonging the treatment (up to 24 h), the simultaneous increase in *V*_ultra_ and decrease in *V*_meso_ (Table 3) and the mesopore volume curves (Figure 2b) indicate that the microporous structure reconstruction takes place at the expense of both small (φ < 3.3 nm) and large (φ > 5 nm) mesopores. After 24 h, the only mesopores still present are those formed around the CTAB aggregates, which are already completely formed after 6 h. These results suggest that during a 6 h NaOH+CTAB treatment the alkaline attack on the microporous structure prevails over the structure reconstruction induced by the hydrothermal conditions, which, however, is already complete around the CTAB aggregates. For longer treatments, reconstruction of the microporous structure further proceeds (*V*_ultra_ increases, *V*_meso_ decreases), without changing the amount of the 3.3–5 nm pores, with no more CTAB aggregates being available.

Thus, N_2_ adsorption results indicate that in order to obtain a hierarchical Y zeolite with significant supermicropore and mesopore contributions, an intermediate duration (6–12 h) of the surfactant-mediated hydrothermal alkaline treatment should be selected, in spite of the low crystallinity degree observed by XRD. Indeed, it is likely that the low *C*_XRD_ values obtained for Y_C6 and Y_C12 (37 and 50%, respectively) are actually due to the much higher mesopore volume (0.07 and 0.06 cm^3^ g^−1^) they possess with respect to the reference NaY (0.01 cm^3^ g^−1^). In particular, if the aim is a hierarchical Y zeolite with a wide range of mesopore widths, resulting in a high total mesopore volume, the treatment should last 6 h. Only in the case the presence of mesopore widths limited to the 3.3–5 nm range is required, the treatment should be prolonged up to 24 h.

**Figure 3:**
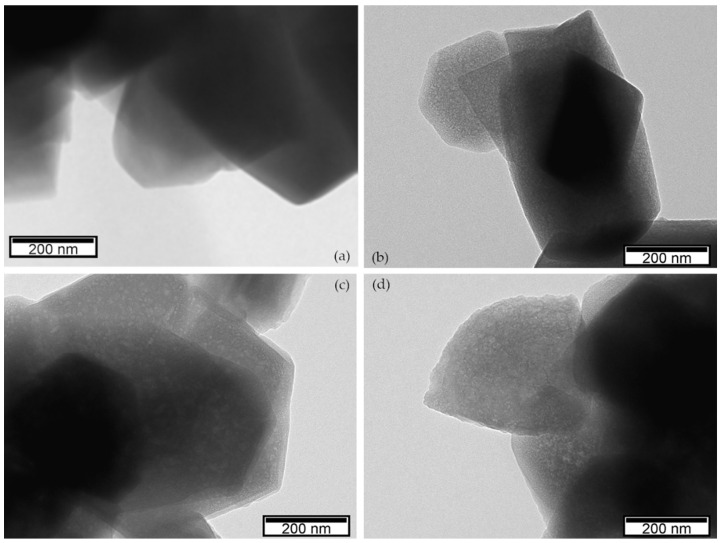
TEM micrographs of the Y zeolites: (**a**) NaY; (**b**) Y_C2; (**c**) Y_C6; (**d**) Y_C12.

The differences in the structural and textural features of the commercial and hierarchical Y materials are also confirmed by the TEM images. Micrographs of the commercial and the hierarchical Y_C2, Y_C6, and Y_C12 samples (Figure 3) show that, compared to the smooth surface of the NaY zeolite (Figure 3a), the external surface of the zeolite particles is rougher and damaged after the alkaline treatment, in agreement with the crystallinity loss observed for the hierarchical samples. Another interesting feature is the presence of several lighter spots in the micrographs of the hierarchical samples, not observed in the NaY, which are attributed, according to the literature, to the enlargement of the pores determined by the surfactant-mediated hydrothermal treatment [19].

**Figure 4:**
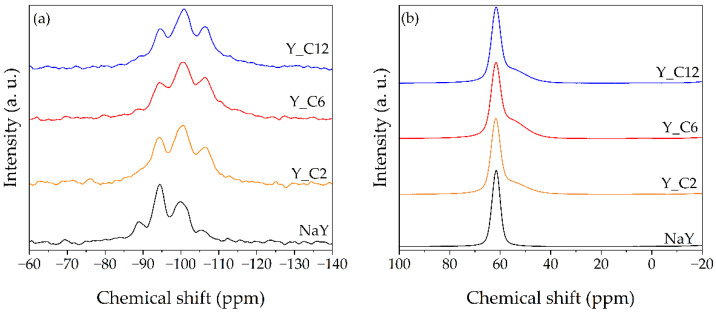
(**a**) ^29^Si MAS-NMR and (**b**) ^27^Al MAS-NMR spectra of commercial and hierarchical Y zeolites.

The ^29^Si MAS-NMR spectra of selected Y zeolites, reported in Figure 4a, show remarkable differences between the NaY and the hierarchical samples. In the former a total of four peaks are observed: a very weak peak at −105 ppm, attributed to the Si(OSi)_4_ crystallographic environments (Q^4^ sites), an intense broad peak at −100 ppm, attributed to Si(X)(OSi)_3_ environment (Q^3^ sites, with X = OH or OAl), another intense peak at −94 ppm, corresponding to the Si(X)_2_(OSi)_2_ environment (Q^2^ sites), and finally a weak signal at −89 ppm, assigned to the Si(X)_3_(OSi) tetrahedra (Q^1^ sites) [23,24]. In the spectra of the hierarchical samples the two peaks at −89 ppm and −94 ppm, corresponding to the Q^1^ and Q^2^ sites, respectively, are less intense compared to the NaY zeolite, whereas the peaks at −105 ppm and −100 ppm become much more intense. No signals ascribable to Q^0^ sites (Si(X)_4_) can be observed. The relative amounts of the Q^n^ sites, reported in Table 4, show that the effect of the alkaline treatment is the reduction of the Q^1^ and Q^2^ sites, which are replaced by the Q^3^ and Q^4^ sites. The increase in the Q^4^ amount, together with the decrease in the Q^1^ and Q^2^, leads to an increase in the calculated Si/Al ratio, as reported in Table 4. Such values and their trend are in agreement with the ICP-OES analysis results and further suggest that the acidic pretreatment before the hydrothermal step determines a partial removal of the Al from the original structure. The remarkable increase in the Q^3^ amount can be attributed to a higher presence of SiOH groups [25], which could be reasonably identified as the silanol groups present on the formed mesopore walls.

The ^27^Al MAS-NMR spectra of the NaY and hierarchical Y zeolites are reported in Figure 4b. All the samples show an intense peak at 63 ppm, indicating a strong resonance of the tetrahedral Al framework typical of the zeolite structure. All the hierarchical samples present a weak shoulder at *ca.* 50 ppm, which could be attributed to a small four-coordinated extraframework Al contribution [26]. No signals around 0 ppm are observable, indicating the absence of octahedral extraframework Al species. This confirms the removal of the debris formed during the pretreatment.

### 3.2. ZSM-5 Zeolites

For the ZSM-5 zeolite, a two-step pretreatment procedure prior to the hydrothermal surfactant-mediated alkaline treatment has been proposed, consisting first of a basic and subsequently an acidic wash [14,18]. However, the effectiveness of the pretreatment employed was found to be limited [19]. Therefore, at variance with the Y zeolites—for which the pretreatment conditions were already defined and the duration of the NaOH+CTAB treatment was changed—for the present ZSM-5 zeolite, the hydrothermal surfactant-mediated alkaline treatment always lasted 6 h, but solutions of different concentrations were employed in the pretreatment.

From the ICP-OES analysis of the commercial NH_4_ZSM-5 zeolite, a Si/Al molar ratio of 14 was determined (nominal Si/Al value 15, calculated from the SiO_2_/Al_2_O_3_ molar ratio value of 30 provided by the producer). As well as for the Y materials, the ratio was found to be higher (*ca*. 20) after the hydrothermal treatment, probably as a consequence of the pretreatment.

**Figure 5:**
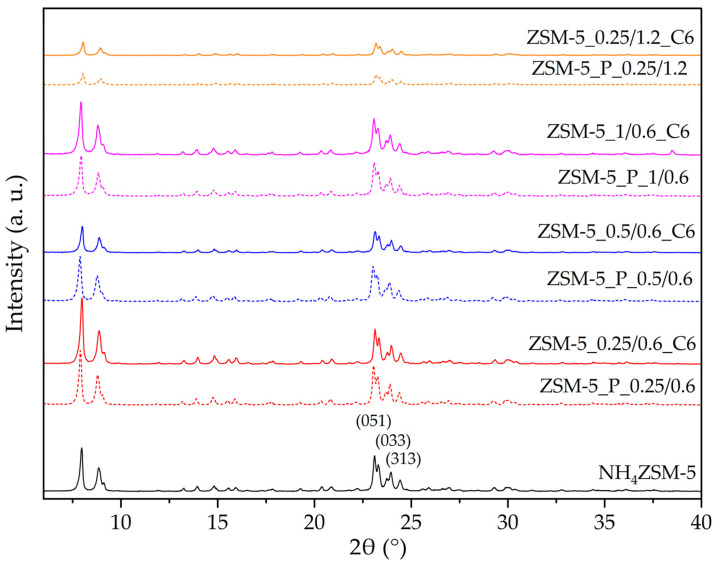
X-ray diffraction patterns of the ZSM-5 zeolites. Miller indexes are attributed according to the IZA Database of Zeolite Structures (http://www.iza-structure.org/databases (accessed on 9 July 2024)).

In Figure 5, the XRD pattern of the NH_4_ZSM-5 zeolite is reported, along with those of the pretreated and the final materials. All the samples show the typical pattern of the MFI zeolite structure. Noteworthily, some significant differences can be observed, which reveal a different effectiveness of the pretreatments in modifying the starting structure and a different effect of the subsequent surfactant-mediated treatment, depending on the pretreated sample on which it is applied. When a 1.2 M H_2_SO_4_ solution is used in the second step of the pretreatment (ZSM-5_P_0.25/1.2), the structure is drastically damaged, and the effect of the subsequent 6 h surfactant-mediated treatment (ZSM-5_0.25/1.2_C6) does not seem significant. If the acid solution concentration is lower (0.6 M), the structure modifications induced by the pretreatment are minor, regardless of the concentration of the NaOH solution used in the first step. However, the final materials obtained after the hydrothermal treatment show some significant structural differences.

The degree of crystallinity was assessed to better describe the effect of both the two-step pretreatment and the hydrothermal treatment on the structure of the zeolite; the results are reported in Table 5. For the ZSM-5 zeolite the same procedure employed for the Y zeolite (*cfr.* 3.1) was applied to the area of the (322), (303), and (133) peaks [21]. The results for the pretreated materials show the effect of the different concentrations of the basic and acidic solutions employed, whereas those for the final materials show how the surfactant-mediated hydrothermal treatment induces the reconstruction of the structure.

After a mild pretreatment (NaOH 0.25 or 0.5 M, H_2_SO_4_ 0.6 M), the crystallinity loss is very low (5%), and the structure does not appear to be damaged enough to be reconstructed by the subsequent 6 h hydrothermal treatment. Actually, during the NaOH+CTAB treatment the crystallinity degree further decreases (Table 5), especially on the ZSM-5_P_0.5/0.6 sample, which suggests that on this pretreated zeolite the destructive effect of NaOH is more important than the structure reconstruction.

When the concentration of the pretreatment NaOH solution is further increased (1 M) a loss in crystallinity of 46 % with respect to the starting ZSM-5 is observed; such a highly damaged structure appears to readily undergo reconstruction upon the NaOH+CTAB treatment, as indicated by the high *C*_XRD_ value (Table 5) for ZSM-5_1/0.6_C6. These results indicate that for the hydrothermal treatment to promote reconstruction, the concentration of the basic solution employed in the pretreatment needs to be high enough to significantly affect the zeolite structure.

On the other hand, when the acidic step of the pretreatment is stronger (H_2_SO_4_ 1.2 M), the zeolite structure is too severely affected and cannot be reconstructed during the NaOH+CTAB treatment.

These results suggest that, in terms of crystallinity, for the ZSM-5 zeolite the best pretreatment combination is a strong alkaline treatment followed by a mild acidic wash. However, crystallinity cannot be considered the determining characteristic for choosing a hierarchical material. Actually, for the Y zeolite it was found that if a high mesopore volume is required, the materials characterized by a not too high *C*_XRD_ value (i.e., obtained with intermediate treatment times) should be selected. Therefore, the textural features of all the ZSM-5 materials need to be studied.

**Figure 6:**
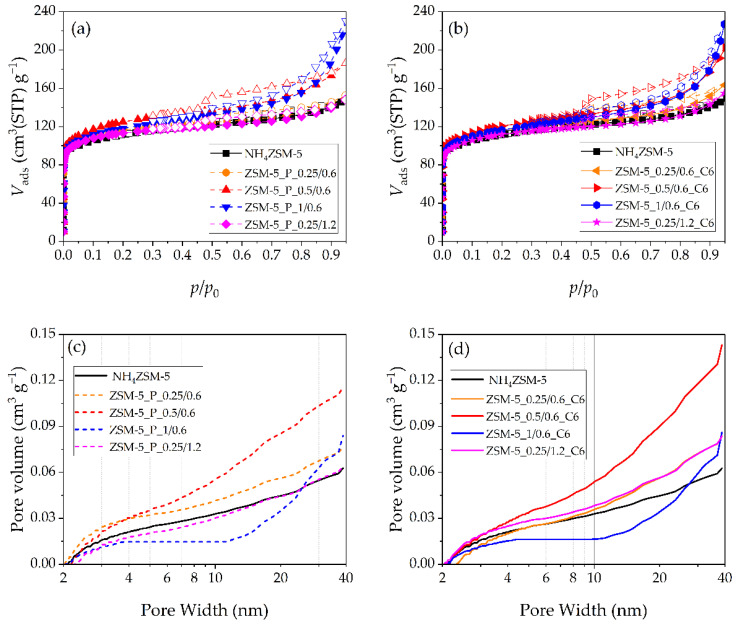
N_2_ physisorption isotherms of (**a**) pretreated ZSM-5_P_*x*/*y* and (**b**) final ZSM-5_*x*/*y*_C6 zeolites (adsorption: full symbols, desorption: open symbols); Cumulative mesopore volume curves obtained with the DFT method for (**c**) pretreated ZSM-5_P_*x*/*y* and (**d**) final ZSM-5_*x*/*y*_C6 zeolites. Starting NH_4_ZSM-5 data are also reported.

The N_2_ physisorption isotherms are reported in Figure 6a for the pretreated materials and in Figure 6b for the final materials; in both figures, the isotherms of the starting NH_4_ZSM-5 zeolite are also shown. The corresponding cumulative pore volume curves obtained by applying the DFT method are reported in Figure 6c,d, respectively. The results of the α_s_-method are summarized in Table 5, in terms of the volume of ultramicropores (φ < 0.7 nm), supermicropores (0.7 nm ≤ φ ≤ 2 nm), and mesopores (φ > 2 nm).

All the ZSM-5 materials show a variety of composite isotherms of type I+II, with different shapes of the hysteresis loop. The isotherms of the zeolites obtained after the mildest pretreatment (ZSM-5_P_0.25/0.6) or after the harsh acidic step (ZSM-5_P_0.25/1.2), as well as when submitted to the subsequent CTAB-mediated 6 h treatment (ZSM-5_0.25/0.6_C6 and ZSM-5_0.25/1.2_C6), are very similar to that of the starting zeolite. The results of the α_s_-method (Table 5) also show that both pretreated materials are very similar to the NH_4_ZSM-5 zeolite. As for the hydrothermally treated samples, together with the buildup of some mesoporosity, the simultaneous decrease in *V*_ultra_ and increase in *V*_super_ can be noticed. The pore volume curves obtained by the DFT method (Figure 6c,d) also indicate similar features in the mesopore range of the starting NH_4_ZSM-5 zeolite, the two pretreated samples, and the two final materials. Therefore, when the first step is performed with a dilute basic solution, the effect of the pretreatment on the textural features is minor whatever the H_2_SO_4_ solution concentration, although the extent of the structure modification depended on the concentration of the acid solution (*cfr*. XRD results). The subsequent NaOH+CTAB treatment does not produce any significant change in the two pretreated samples, in terms of both structural and textural features. As expected, the surfactant-mediated alkaline treatment is not effective if the pore system has not been properly fragilized by the pretreatment.

The isotherms of the materials pretreated with 0.5 or 1 M NaOH (and 0.6 M H_2_SO_4_) solutions exhibit more pronounced hysteresis loops both before (Figure 6a) and after (Figure 6b) the CTAB-mediated 6 h treatment. Appling the α_s_-method, the formation of mesopores in all these materials, while the micropore features do not change, is evident (Table 5). The mesopore volume curves obtained with the DFT method (Figure 6c) show a much higher contribution of large mesopores for ZSM-5_P_0.5/0.6 and ZSM-5_P_1/0.6 compared to the other materials. However, the distribution of the mesopore volume in the two samples is different, with the pores in the range 4-11 nm being practically absent in ZSM-5_P_1/0.6. The NaOH+CTAB treatment produces no significant modifications in the textural properties of the two pretreated materials, despite the totally different effect on the crystallinity degree (Table 5).

Thus, all the pretreatment conditions tested on the ZSM-5 zeolite were inefficient in damaging the original structure in a way that allows the reconstruction to occur around the CTAB aggregates. In fact, a concentrated acidic solution does not modify the textural properties; on the contrary, it significantly damages the zeolite structure, which is not reconstructed during the hydrothermal treatment. The effect of the first step of the pretreatment increases with the base concentration, but it seems to be not enough for letting the surfactant aggregates diffuse within the damaged structure. Even when the hydrothermal treatment results in a structure reconstruction (ZSM-5_1/0.6_C6), the mesoporosity does not increase with respect to the corresponding pretreated material.

**Figure 7:**
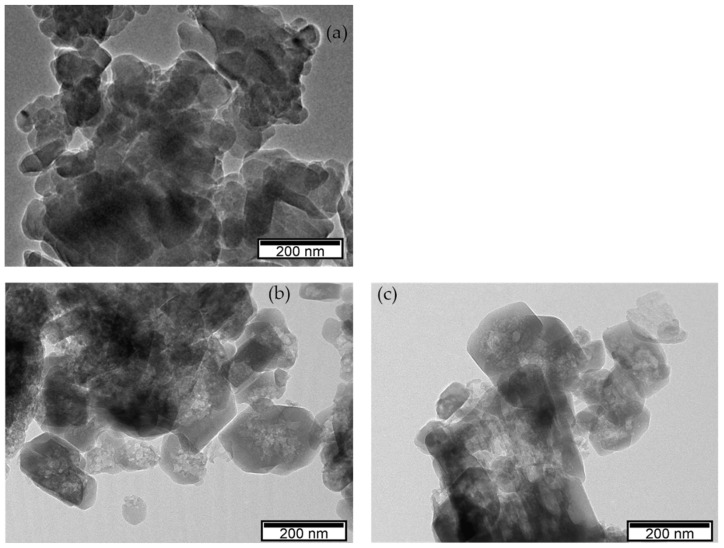
TEM micrographs of the ZSM-5 zeolites: (**a**) NH_4_ZSM-5; (**b**) ZSM-5_P_1/0.6; (**c**) ZSM-5_1/0.6_C6.

The presence of the lighter spots in the TEM micrographs of the pretreated ZSM-5_P_1/0.6 and the final ZSM-5_1/0.6_C6 zeolites (Figure 7) confirms that the enlargement of the pores and the formation of wide mesopores is mostly caused by the pretreatment rather than by the surfactant-mediated treatment. Therefore, if the aim is a hierarchical material with a specific mesopore range, the surfactant-mediated treatment is unsuitable for the ZSM-5 zeolite (at least, after the pretreatments tested). However, if unspecific (i.e., random in size and shape) mesoporosity is pursued, the two-step pretreatment is sufficient, provided that the concentration of the acid solution is not too high and that of the basic solution is properly selected.

**Figure 8:**
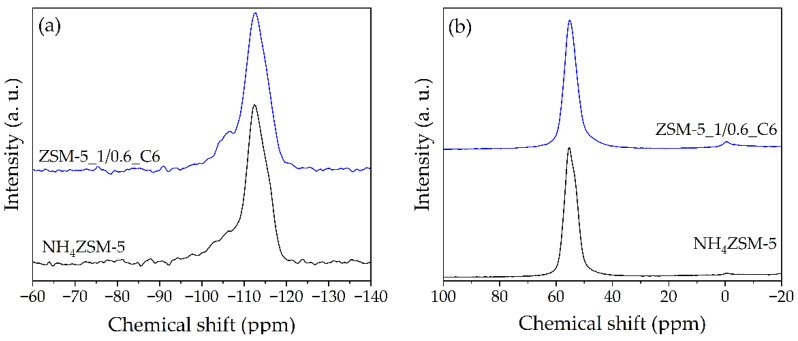
(**a**) ^29^Si MAS-NMR and (**b**) ^27^Al MAS-NMR spectra of NH_4_ZSM-5 and ZSM-5_1/0.6_C6 zeolites.

**Figure 9:**
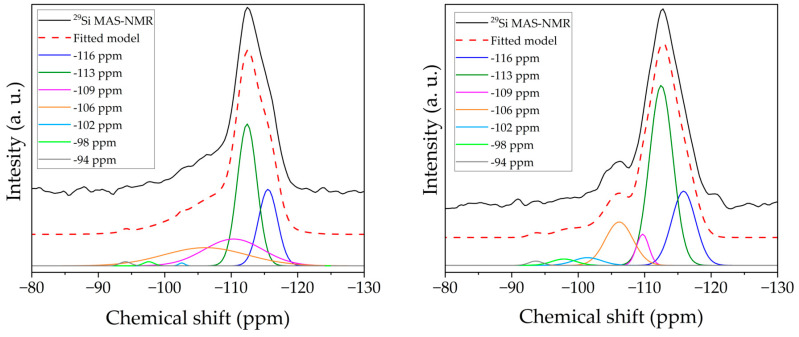
Deconvolution of the ^29^Si MAS-NMR spectra of (**a**) NH_4_ZSM-5 and (**b**) ZSM-5_1/0.6_C6.

The ^29^Si MAS-NMR spectra of the starting NH_4_ZSM-5 and the selected hierarchical ZSM-5_1/0.6_C6 zeolites, reported in Figure 8a, display a poor resolution, typical of this highly siliceous zeolitic framework [27]. For both the ZSM-5 and the hierarchical zeolites, a very intense and broad peak from *ca.* −100 ppm to −119 ppm with a maximum at −113 ppm is observed. For the resolution of these complex spectra, a deconvolution method was involved [28]. A total of seven Gaussian components were used and the results are shown in detail in Figure 9. In accordance with [28], the resonances at −116 and −113 ppm were assigned to Q^4^ sites; the bands centered at −109 and −106 ppm were attributed to Q^3^ sites; the signals at −102, −98, and −94 ppm were ascribed to Q^2^, Q^1^, and Q^0^ sites, respectively. The Q^n^ distribution is reported in Table 6, together with the Si/Al ratio values, which are in very good agreement with those obtained by ICP-OES analysis. As already observed for the Y zeolites, the increase in the Q^4^ sites and the corresponding increase in the Si/Al ratio suggest that the pretreatment induces a partial removal of the Al from the original structure.

The ^27^Al MAS-NMR spectra of the commercial and hierarchical ZSM-5_1/0.6_C6 zeolites are reported in Figure 8b. As observed in the case of the Y samples (*cfr.* Figure 4b), the spectra show an intense resonance of the tetrahedral Al framework at 63 ppm. Both the samples present a very weak signal at *ca.* 0 ppm, which is attributed to octahedral extraframework Al. In the case of the hierarchical zeolite the intensity of this last signal is slightly higher, probably due to a not complete removal of the debris formed during the pretreatment.

### 3.3. Comparison between Y and ZSM-5 Zeolites

Besides the studies performed within each series, it is important to note that the effect of the NaOH+CTAB hydrothermal treatment on the two zeolite structures is different. Ordered intraparticle mesoporosity is obtained in the Y zeolites, provided that the treatment is long enough to efficiently damage the structure before it is reconstructed around the surfactant aggregates, but not so long to completely destroy it. On the other hand, the mesopores formed in the ZSM-5 zeolites are not characterized by a typical width and are mainly formed during the two-step treatment. Such differences are certainly due to the completely different porous structure of the two zeolites, together with the different Si/Al ratios and the different pretreatments they undergo.

After a mild acidic pretreatment, the large micropores and the supercages typical of the Y zeolite, further enlarged by NaOH, allow the diffusion of CTAB and the formation of narrow intraparticle mesopores (3.3–5 nm) around the surfactant aggregates during the hydrothermal treatment.

The high-silica ZSM-5 requires a two-step pretreatment, upon which—if the basic solution is concentrated enough and the acidic solution is not too concentrated—disordered mesopores are formed. The subsequent hydrothermal treatment induces, depending on the *C*_XRD_ of the pretreated material (Table 5), either reconstruction or further destruction of the structure, but without significant changes in the porous features. Most likely, the narrow micropores typical of the ZSM-5 zeolite are not sufficiently enlarged during the hydrothermal treatment, at least with the present conditions and after the pretreatments employed.

On the whole, hierarchical materials can be obtained for both systems, but the pretreatment and treatment conditions need to be selected depending on the starting zeolite and on the type of mesoporosity needed, avoiding unnecessary steps.

If only mesopores in the 3.3–5 nm range are required in a Y zeolite, the CTAB-mediated hydrothermal treatment should last 24 h. If a higher mesopore volume (0.07 cm^3^ g^−1^), related to the presence of smaller and larger pores, is appreciated, or at least acceptable, the ideal treatment duration is 6 h.

As for the ZSM-5, it appears that with the treatments employed only a disordered mesoporosity can be induced and that for this purpose the sole two-step pretreatment is sufficient. However, it cannot be excluded that with a different pretreatment (in particular, a higher concentration of the base) and/or a modified hydrothermal treatment the formation of the surfactant aggregates could be exploited in a “real” surfactant-mediated destructive–reconstructive alkaline hydrothermal treatment.

## 4. Conclusions

The production of hierarchical Y and ZSM-5 zeolites was performed using the surfactant-mediated hydrothermal alkaline method, using CTAB and NaOH.

In the case of the Y zeolites, it was found that the effectiveness of the NaOH+CTAB hydrothermal treatment, preceded by a mild acidic pretreatment, depends on its duration. If the treatment is too short (2 h) the formation of mesopores is at an initial stage, whereas if it is too long (48 h) the zeolite structure is completely destroyed. For intermediate treatment times (6–12 h) relatively high mesopore volumes (0.06–0.07 cm^3^ g^−1^) are obtained, resulting not only from the 3.3–5 nm pores obtained through the structure reconstruction around the surfactant aggregates but also from both smaller and larger mesopores. After a 24 h treatment mesopore volume becomes lower (0.04 cm^3^ g^−1^) but the 3.3–5 nm pores are preserved.

For ZSM-5 zeolites, in the present conditions, only a disordered mesoporosity could be induced. This was already present in the samples obtained by the two-step pretreatment, the subsequent hydrothermal treatment not causing significant changes in the porous features. The effect of the concentration of the basic and acidic solutions employed in the pretreatment was studied. It was found that, in order to develop a significant mesopore volume, the acid solution used in the second step should not be too concentrated, whereas in the first step the base concentration needs to be at least 0.5 M. However, even when a significant mesoporosity was induced (for ZSM-5_P_1/0.6 *V*_meso_ = 0.23 cm^3^ g^−1^), the reconstruction around the CTAB aggregates during the hydrothermal treatment was not effectively exploited.

The present results clearly indicate that, depending on the type of mesoporosity required and on the zeolite structure to be modified, pretreatment and treatment conditions need to be finely tailored. For the Y zeolite, intermediate treatment times (e.g., in the range of 4–8 h), as well as the effect of other parameters, such as stirring or using larger autoclaves, could be further investigated. For the ZSM-5 zeolite, a more effective pretreatment could be sought (e.g., using a more concentrated base solution). Nonetheless, given the hierarchical porous structure, some of the present samples appear to be suitable for testing in catalytic applications.

## Figures and Tables

**Table 1:** Operating parameters in the preparation of Y zeolites by modification of NaY.

Sample	Pretreatment			Hydrothermal Treatment			
	Citric Acid Concentration (wt%)	*T* (°C)	*t* (h)	NaOH Concentration (M)	CTAB Concentration (M)	*T* (°C)	*t* (h)
Y_P	10	25	17	-	-	-	-
Y_C2	10	25	17	0.37	0.03	150	2
Y_C6	10	25	17	0.37	0.03	150	6
Y_C12	10	25	17	0.37	0.03	150	12
Y_C24	10	25	17	0.37	0.03	150	24
Y_C48	10	25	17	0.37	0.03	150	48

**Table 2:** Operating parameters in the preparation of ZSM-5 zeolites by modification of NH_4_ZSM-5.

Sample	Two-Step Pretreatment						Hydrothermal Treatment			
	First Step			Second Step						
	NaOH Concentration (M)	*T* (°C)	*t* (h)	H_2_SO_4_ Concentration (M)	*T* (°C)	*t* (h)	NaOH Concentration (M)	CTAB Concentration (M)	*T* (°C)	*t* (h)
ZSM-5_P_0.25/0.6	0.25	80	1	0.6	80	3	-	-	-	-
ZSM-5_P_0.5/0.6	0.5	80	1	0.6	80	3	-	-	-	-
ZSM-5_P_1/0.6	1	80	1	0.6	80	3	-	-	-	-
ZSM-5_P_0.25/1.2	0.25	80	1	1.2	80	3	-	-	-	-
ZSM-5_0.25/0.6_C6	0.25	80	1	0.6	80	3	0.37	0.03	150	6
ZSM-5_0.5/0.6_C6	0.5	80	1	0.6	80	3	0.37	0.03	150	6
ZSM-5_1/0.6_C6	1	80	1	0.6	80	3	0.37	0.03	150	6
ZSM-5_0.25/1.2_C6	0.25	80	1	1.2	80	3	0.37	0.03	150	6

**Table 3:** Structural and textural properties of the Y zeolites.

Sample	*C*_XRD_ ^a^ (%)	*V*_ultra_ ^b^ (cm^3^ g^−1^)	*V*_super_ ^b^ (cm^3^ g^−1^)	*V*_meso_ ^b^ (cm^3^ g^−1^)
NaY	100	0.33	0.01	0.01
Y_P	72	0.16	0.01	0.02
Y_C2	90	0.21	0.07	0.05
Y_C6	37	0.16	0.08	0.07
Y_C12	50	0.19	0.07	0.06
Y_C24	91	0.20	0.07	0.04

^a^ Assessed from XRD patterns. ^b^ Assessed by the application of the α_s_-method to the N_2_ physisorption isotherms.

**Table 4:** Q^n^ site percentage distribution and Si/Al molar ratio obtained from the ^29^Si MAS-NMR spectra of the Y zeolites.

Sample	Q^n^ Site Distribution (%)				Si/Al
	Q^1^	Q^2^	Q^3^	Q^4^	
NaY	13	42	37	8	2.5
Y_C2	0	32	44	25	3.7
Y_C6	4	24	45	27	3.8
Y_C12	4	23	46	27	3.8

**Table 5:** Structural and textural properties of the ZSM-5 zeolites.

Sample	*C*_XRD_ ^a^ (%)	*V*_ultra_ ^b^ (cm^3^ g^−1^)	*V*_super_ ^b^ (cm^3^ g^−1^)	*V*_meso_ ^b^ (cm^3^ g^−1^)
NH_4_ZSM-5	100	0.13	0.03	0.08
ZSM-5_P_0.25/0.6	95	0.13	0.03	0.08
ZSM-5_P_0.5/0.6	95	0.13	0.02	0.14
ZSM-5_P_1/0.6	54	0.12	0.01	0.23
ZSM-5_P_0.25/1.2	30	0.12	0.03	0.08
ZSM-5_0.25/0.6_C6	91	0.10	0.05	0.11
ZSM-5_0.5/0.6_C6	69	0.13	0.01	0.18
ZSM-5_1/0.6_C6	91	0.11	0.02	0.22
ZSM-5_0.25/1.2_C6	32	0.09	0.06	0.10

^a^ Assessed from XRD patterns. ^b^ Assessed by the application of the α_s_-method to the N_2_ physisorption isotherms.

**Table 6:** Q^n^ site percentage distribution and Si/Al molar ratio obtained from ^29^Si MAS-NMR spectra of the ZSM-5 zeolites.

Sample	Q^n^ Site Distribution (%)					Si/Al
	Q^0^	Q^1^	Q^2^	Q^3^	Q^4^	
NH_4_ZSM-5	2	4	3	27	64	15
ZSM-5_1/0.6_C6	1	2	2	20	75	20

## Data Availability

Data are contained within the article.
